# Communication of prognosis to patient with PSP and family

**DOI:** 10.1002/alz70857_101939

**Published:** 2025-12-25

**Authors:** Juan‐Camilo Vargas‐González, Ignacio Flores, Carmela Tartaglia, Indira Ruth Garcia Cordero, Alonso Morales‐Rivero, Raul Medina Rioja, Blas Couto

**Affiliations:** ^1^ Memory Clinic, Toronto Western Hospital, University Health Network, Toronto, ON, Canada; ^2^ University Health Network, Toronto, ON, Canada; ^3^ Neuroscience Institute, Favaloro Foundation, Buenos Aires, Buenos Aires, Argentina; ^4^ Rossy PSP Program, University Health Network and the University of Toronto, Toronto, ON, Canada; ^5^ Canadian Concussion Centre, Toronto Western Hospital, University Health Network, Toronto, ON, Canada; ^6^ Division of Neurology, Toronto Western Hospital, University Health Network Memory Clinic, Toronto, ON, Canada; ^7^ Universidad de San Andres, Victoria, Buenos Aires, Argentina; ^8^ Toronto Western Hospital, Memory Clinic, Toronto, ON, Canada; ^9^ Instituto Nacional de Neurología y Neurocirugía “Manuel Velasco Suárez”, Mexico, DF, Mexico; ^10^ Laboratory of Progressive Neuromotor and Neurobehavioral Disorders at Instituto de Neurociencia Cognitiva y Traslacional (INCYT), CABA, Buenos Aires, Argentina; ^11^ Unidad de Movimientos Anormales, Institute of Neuroscience of the Fundacion Favaloro, CABA, Buenos Aires, Argentina

## Abstract

The prognosis of progressive supranuclear palsy (PSP) and corticobasal syndrome (CBS) involves a survival shorter than 10‐years since symptom onset with development of marked disability. Patients will often progress to require increased assisstance with daily living activities, and symptomatic therapies with partial effectiveness that lead to experiencing decreased quality of life. Speech difficulties and swallowing problems dictate the moderate to severe stages of the disease course adding complexity to the daily care of patients with PSP and CBS. Altogether, these create a huge burden that impacts directly to the patient family and caregivers. Communicating prognosis at each timepoint of the disease is crucial for care planning and making future life decisions. In this session, we will provide clinicians with information and skills to communicate prognosis of tauopathies with an accertive and patient‐centered approach.

